# Heat Transfer Enhancement of Laminar Nanofluids Flow in a Circular Tube Fitted with Parabolic-Cut Twisted Tape Inserts

**DOI:** 10.1155/2014/543231

**Published:** 2014-01-30

**Authors:** Sami D. Salman, Abdul Amir H. Kadhum, Mohd S. Takriff, Abu Bakar Mohamad

**Affiliations:** ^1^Department of Chemical and Process Engineering, Faculty of Engineering and Built Environment, Universiti Kebangsaan Malaysia, 43600 Bangi, Selangor, Malaysia; ^2^Biochemical Engineering Department, Al-khwarizmi College of Engineering, University of Baghdad, Baghdad 47024, Iraq

## Abstract

Numerical investigation has been carried out on heat transfer and friction factor characteristics of copper-water nanofluid flow in a constant heat-fluxed tube with the existence of new configuration of vortex generator using Computational Fluid Dynamics (CFD) simulation. Two types of swirl flow generator: Classical twisted tape (CTT) and Parabolic-cut twisted tape (PCT) with a different twist ratio (*y* = 2.93, 3.91 and 4.89) and different cut depth (*w* = 0.5, 1.0 and 1.5 cm) with 2% and 4% volume concentration of CuO nanofluid were used for simulation. The effect of different parameters such as flow Reynolds number, twist ratio, cut depth and nanofluid were considered. The results show that the enhancement of heat transfer rate and the friction factor induced by the Classical (CTT) and Parabolic-cut (PCT) inserts increases with twist ratio and cut depth decreases. The results also revealed that the heat transfer enhancement increases with an increase in the volume fraction of the CuO nanoparticle. Furthermore, the twisted tape with twist ratio (*y* = 2.93) and cut depth *w* = 0.5 cm offered 10% enhancement of the average Nusselt number with significant increases in friction factor than those of Classical twisted tape.

## 1. Introduction

The applications of heat transfer augmentation techniques can significantly increase the performance of heat exchanger, leading to the reduction of heat exchanger size as well as operating cost. The augmentation is classified into three main techniques, namely, active, passive, and compound. The active techniques require an external force such as electric field, acoustic, or surface vibration. The passive technique involves fluid additives, special surface geometries, or swirl flow devices, that is, Twisted tape inserts. On the other hand, the compound techniques are made by a combination between two or more passive and/or active techniques. Several experimental studies on heat transfer augmentation techniques using twisted tape have been reported in the literature [1–15], as well as theoretical studies using CFD modeling. Kharat et al. [16] developed the new correlation of heat transfer coefficient between concentric helical coils of a heat exchanger which depended on experimental work and CFD simulation. Fluent 6.3.26 has been used to improve the heat transfer coefficient correlation for the flue gas side to optimize the gap between concentric coils. Pathipakka and Sivashanmugam [17] proposed CFD simulation of the heat transfer characteristics of a Al_2_O_3_ nanofluid in a circular tube fitted with helical twist inserts under constant heat flux using Fluent version 6.3.26 in a laminar flow. The Al_2_O_3_ nanoparticles in water at different concentrations (0.5%, 1.0%, and 1.5%) and helical twist inserts with different twist ratios (*y* = 2.93, 3.91, and 4.89) were used for the simulation. The data obtained by simulation was compared with the literature value of water for plain tube helical tape inserts. Salman et al. [18] reports an application of a mathematical model of the heat transfer enhancement and friction factor characteristics of water in constant heat-fluxed tube fitted with elliptical cut twisted tape inserts using FLUENT version 6.3.26. Two types of swirl flow generator: Classical and elliptical cut twisted tape with twist ratio (*y* = 2.93, 3.91, 4.89) and different cut depths (*w* = 0.5, 1, 1.5 cm) were used for simulation. The results elaborated that the enhancement of heat transfer rate and the friction factor induced by elliptical cut twisted tape inserts increase with Reynolds number and decrease with twist ratio. In addition, the results show that the elliptical cut twisted tape with twist ratio (*y* = 2.93) and cut depth (*w*= 0.5 cm) offered higher heat transfer rate with significant increases in friction factor. Salman et al. [19] numerically studied heat transfer of water in a uniformly heated circular tube fitted with V-cut twisted tape inserts in laminar flow using FLUENT version 6.3.26. Classical and elliptical cut twisted tape with twist ratio (*y* = 2.93, 3.91, 4.89) and different cut depths (*w* = 0.5, 1, 1.5 cm) were employed for the simulation. The results show that the V-cut twisted tape with twist ratio (*y* = 2.93) and cut depth (*w* = 0.5 cm) present a maximum heat transfer rate with significant increases in friction factor.

In the present work, a numerical investigation of heat transfer enhancement in a tube induced by new configuration of vortex generator (Parabolic-cut twist tape) with 2% and 4% volume fractions of CuO nanofluid is reported using CFD simulation. The result obtained by this configuration offered about 10% enhancement of the Nusselt number with significant increases in friction factor than those of Classical twisted tape.

## 2. Technical Details

### 2.1. Physical Models

**Figure 1:**
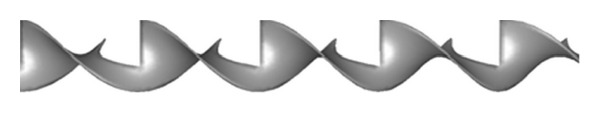
Parabolic-cut twisted insert.

The configuration of the Parabolic-cut twisted tape (PCT) insert is shown in Figure 1. Aluminium tape of 0.08 cm thickness and 2.45 cm width is uniformly winding over a length of 7.5, 10, and 12.5 cm to produce twist ratios of 2.93, 3.91, and 4.89. The twist ratio “*y*” is defined as the ratio of the length of one full twist (360°) to the tape width. Three cut depth (*w* = 0.5, 1, and 1. 5 cm) is used for each twisted tape to produce Parabolic-cut twisted tape.

Steel tube with a diameter (*D*) of 2.54 cm and length (*L*) of 180 cm is used as test section, Water and CuO nanoparticles (*dp*= 29 nm) are selected as the working fluid. The thermophysical properties of fluid and materials used for simulation are shown in Tables 1 and 2.

### 2.2. Thermophysical Properties of Nanofluids

The thermophysical properties of nanofluids used in this study were obtained using the following equations [20]:

(1) $$\begin{array}{c} \rho_{\text{nf}}=\left(1-\phi \right)\rho_{f}+\phi \rho_{\text{np}},\\ {\left(\rho Cp\right)}_{\text{nf}}=\left(1-\phi \right){\left(\rho Cp\right)}_{f}+\phi {\left(\rho Cp\right)}_{\text{np}}, \end{array}$$

where  *ρ*_*f*_ and *ρ*_np_ are the mass densities of the based fluid and the solid nanoparticles, *ϕ* nanoparticle volume concentration, and (*ρCp*)_*f*_ and (*ρCp*)_np_ are heat capacities of the based fluid and the solid nanoparticles.

The effective thermal conductivity can be obtained by using the following mean empirical correlation [20]:

(2) $$\begin{array}{c} K_{\text{eff}}=K_{\text{Static}}+K_{\text{Brownian}},\\ K_{\text{Static}}=K_{f}\left[\frac{\left(K_{\text{np}}+2K_{f}\right)-2\phi \left(K_{f}-K_{\text{np}}\right)}{\left(K_{\text{np}}+2K_{f}\right)+\phi \left(K_{f}+K_{\text{np}}\right)}\right],\\ K_{\text{Brownian}}=5*10^{4}\beta \phi \rho_{f}Cp_{f}\sqrt{\frac{KT}{2\rho_{\text{np}}R_{\text{np}}}}f\left(T,\phi \right), \end{array}$$

where  *β* = 9.881(100*ϕ*)^−0.9446^ for CuO nanoparticle,  *k* = 1.3807∗10^−23^ J/K (Boltzmann constant), and *f*(*T*, *ϕ*) = (2.8217∗10^−2^ *ϕ* + 3.917∗10^−3^)(*T*/*T*_0_) + (−3.669∗10^−2^ *ϕ* − 3.391123∗10^−3^).

The effective viscosity can be obtained by using the following mean empirical correlation [21]:

(3) $$\begin{array}{c} \mu_{\text{eff}}=\mu_{f}*\frac{1}{\left(1-34.87{\left(dp/df\right)}^{-0.3}*\phi^{1.03}\right)},\\ df=\left[\frac{6M}{N\pi \rho_{f_{0}}}\right], \end{array}$$

where *M* is the molecular weight of base fluid, *N* is the Avogadro number = 6.022*∗*10^23^ mol^−1^, and *ρ*_*f*_0__ is the mass density of the based fluid calculated at temperature *T*_0_ = 293 K.

## 3. CFD Simulation

### 3.1. Geometry Creation and Grid Arrangement

**Figure 2:**
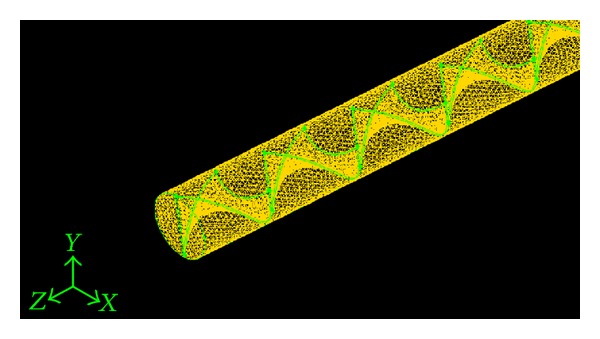
Grid of Parabolic-cut twisted tape (PCT) insert.

The geometry and the gird (mesh) were generated using GAMBIT and FLUENT as CFD solver to handle this grid. The geometry of twist tape inserts is made by winding uniformly a strip of 0.08 cm thickness and 2.45 cm width using a perpendicular sweep face option. The angle of twist is 360° over a length of 7.5, 10, and 12.5 cm to produce the twist ratios in 2.93, 3.91, and 4.89. The sweeping face has been used over the entire length of 1800 mm. The twist tape insert is subtracted from cylindrical volume to obtain the required fluid domain. Different types of meshing elements are available to mesh the volume, but tetrahedral/hybrid and T Grid type elements are the best option in case of irregular shapes. The grid generated for the tube fitted with Parabolic-cut twisted tape insert is shown in Figure 2. The boundary conditions and continuum type for the geometry inlet, outlet, walls, and fluid type were specified. Subsequently, the meshed volume was exported to FLUENT for simulation.

### 3.2. Computational Model

#### 3.2.1. Assumptions

The nanoparticles in the base fluid may be a single phase fluid with thermal equilibrium and with zero relative velocity between the fluid phase and nanoparticles. These assumptions will exactly reflect the behaviour of nanofluid in engineering problems. The problem was investigated for three-dimensional steady state laminar flow using the following model equations with the numerical values of the mass flow rate and constant heat flux given in Table 3.

#### 3.2.2. Continuity Equation for Incompressible Fluid

Consider the following:

(4) $$\begin{array}{c} \frac{\partial p}{\partial t}+\nabla \cdot \left(\rho \vec{\upsilon }\right)=S_{m}. \end{array}$$

#### 3.2.3. Conservation of Momentum

Consider the following:

(5) $$\begin{array}{c} \frac{\partial \upsilon }{\partial t}+\rho \left(\vec{\upsilon }\cdot \nabla \right)\vec{\upsilon }=-\nabla p+\rho \bar{g}+\nabla \cdot \tau_{ij}+\vec{F}. \end{array}$$

#### 3.2.4. Conservation of Energy 

Consider the following:

(6) ρ∂∂t(ρE)+∇·{υ→(ρE+ρ)}  =∇·{Keff∇T−∑hi(τ→eff·υ→)}+Sh.

The following equations are used to calculate the Nusselt number (Nu) and the friction factor (*f*):

(7) Nu=hDK,h=q∙Tw−Tb,f=64Re,Re=ρuDμ,

where *D* is the tube diameter, *h* is the heat transfer coefficient, *k* is the conductivity of water, *q* ^∙^ is the heat flux on the tube, *T*_*w*_ is the tube wall temperature, and *T*_*b*_ is the bulk temperature of water *T*_*b*_ = (*T*_0_ + *T*_*i*_)/2. *ρ* is the density, *μ* is dynamic viscosity, and *u* is the water velocity.

## 4. Results and Discussion

### 4.1. Grid Independence Test and Model Validation

**Figure 3:**
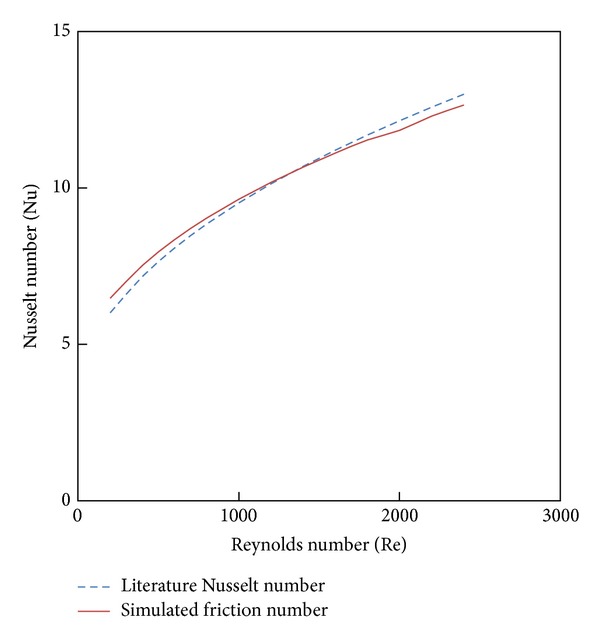
Plain tube simulated Nusselt number versus literature value.

**Figure 4:**
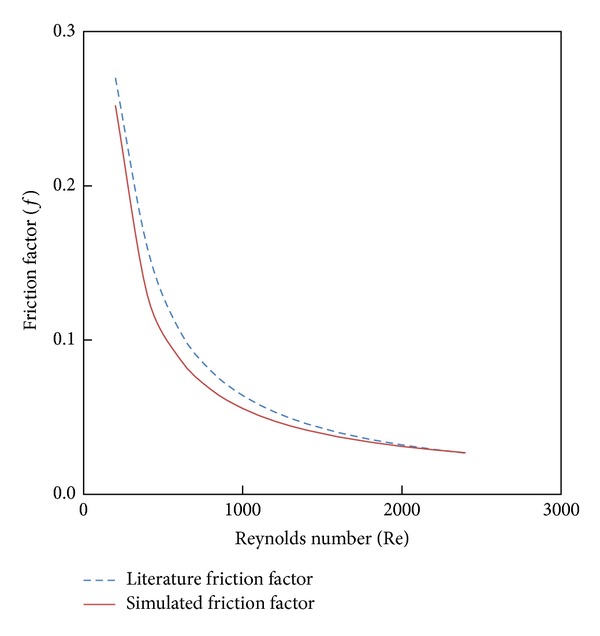
Plain tube simulated friction factor versus literature value.

A grid independence test was tested to evaluate the effects of grid sizes on the simulated results; five mesh volumes were considered at Re = 2000 (401226, 532338, 656404, 727890, and 838278). It is observed that all the five mesh volumes have similar results of the Nusselt number with a percentage error of 0.3%. Hence, a domain with mesh volume of 656404 was chosen to reduce the computational time. Computations data of the Nusselt number and friction factor was first performed for water in a plain tube to validate the model against data developed by Stephan and Preußer correlations [22]. Figures 3 and 4 show the variation of Nusselt number and friction factor with Reynolds number for plain tube. Apparently, the present results reasonably agreed well with the available correlations within ±8% and ±10% for Nusselt number and friction factor, respectively.

### 4.2. Effect of Twist Ratio

**Figure 5:**
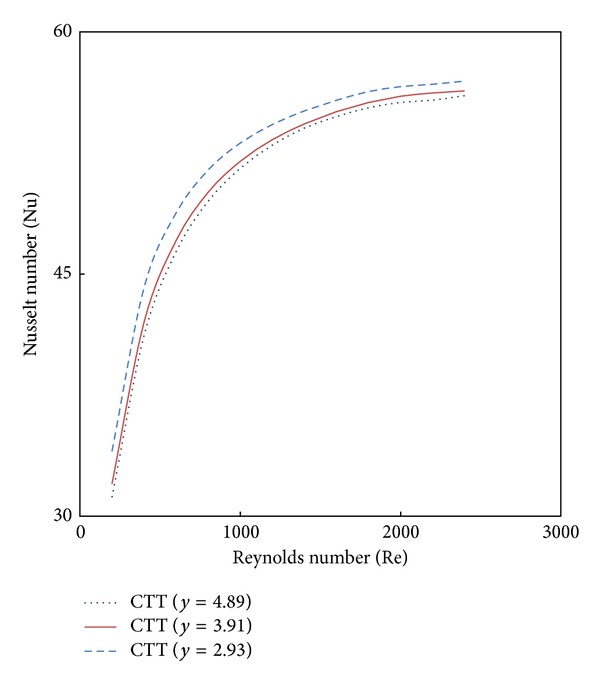
The effect of twist ratio (*y*) on Nusselt Number.

**Figure 6:**
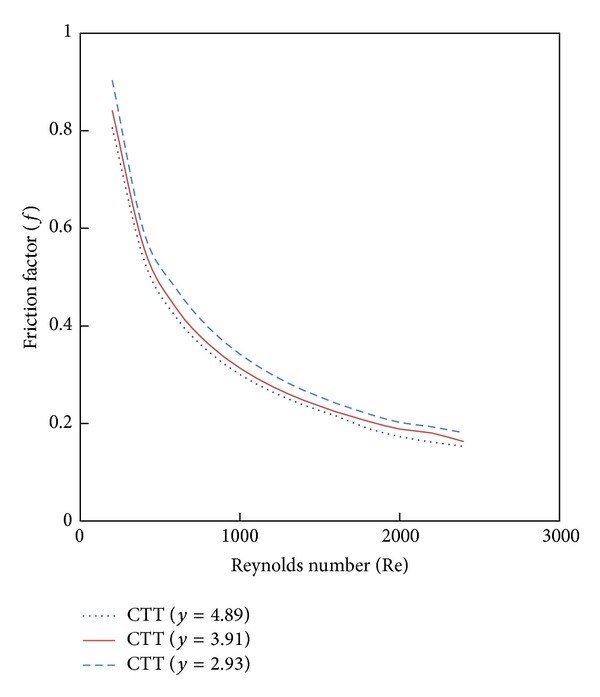
The effect of twist ratio (*y*) on friction factor.

Variation of simulated Nusselt number and friction factor with Reynolds number for water in the presence of classical twisted tape inserts are illustrated in Figures 5 and 6. It is found that the Nusselt number and friction factor in lower twist ratios are higher than those from higher ratio (*y*) across the range of Reynolds number. This means that the lower twist ratio leads to higher tangential contact between the swirling flow and the tube surface.

### 4.3. Effect of Nanofluid Volume Fractions

**Figure 7:**
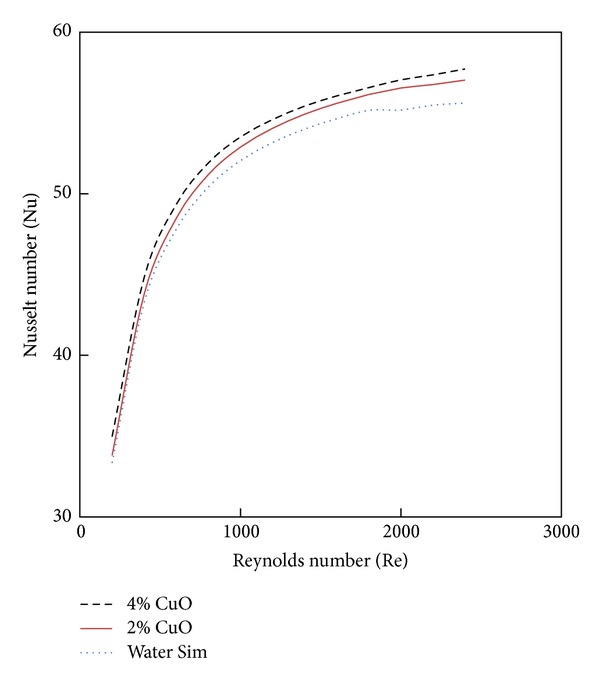
The effect of CuO nanofluid volume concentration on Nusselt number for CCT with twist ratio 2.93.

**Figure 8:**
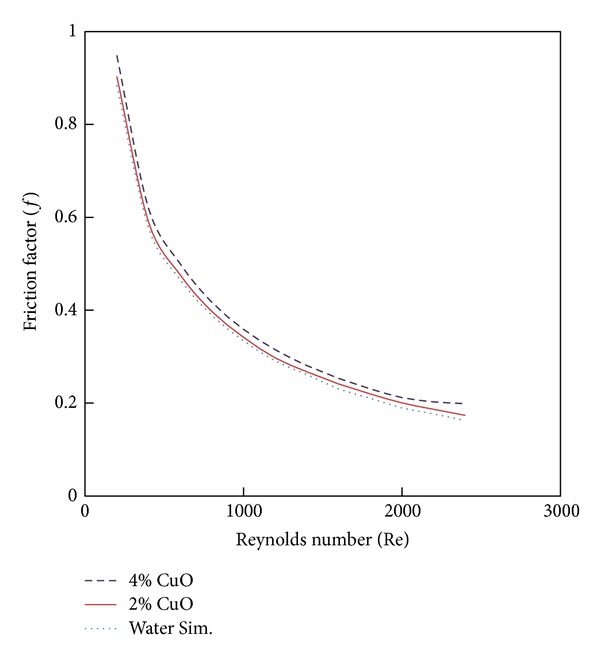
The effect of CuO nanofluid volume concentration on fiction factor for CCT of twist ratio 2.93.

CuO nanoparticles of 2% and 4% volume fraction with different values of Reynolds number are investigated as shown in Figures 7 and 8. From Figure 7, it is clearly noted that the Nusselt number is enhanced with increases of volume fraction of nanoparticles. This means that the volume fraction increases the random movements of the particles and enhances the thermal dispersion of the flow.  Figure 8 shows the friction factor variation with the Reynolds number for different volume fractions of nanoparticles. It is clearly noted that the wall shear stress increases with the increase of the nanoparticles volume fraction.

### 4.4. Effect of Twist Tape Configuration

**Figure 9:**
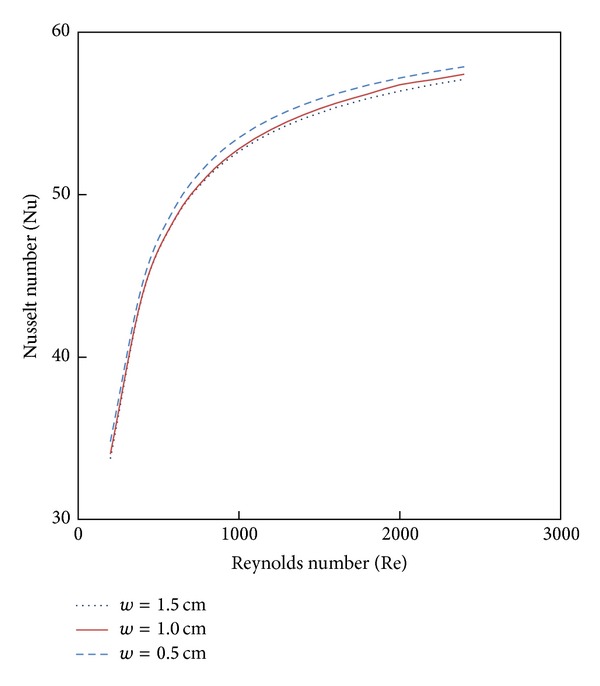
The effect of cut depth 4% CuO nanofluid on Nusselt number.

**Figure 10:**
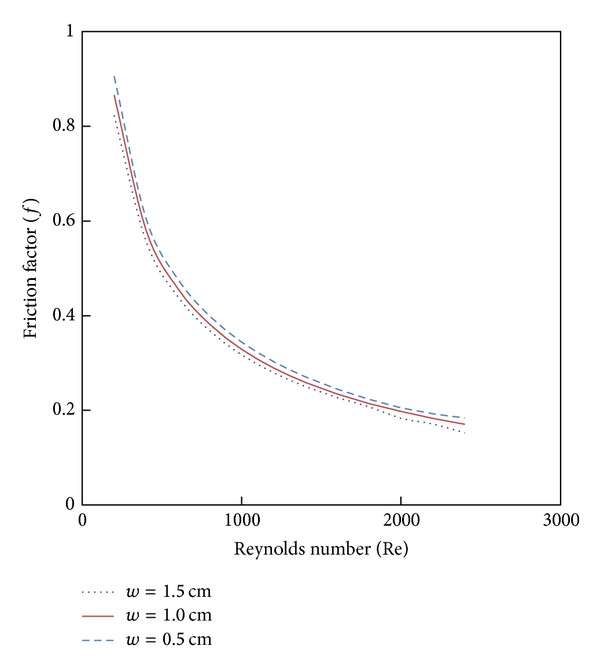
The effect of cut depth with 4% CuO nanofluid on friction factor.

Variation of simulated Nusselt number and friction factor with Reynolds number for the tube fitted Parabolic-cut twist tape with twist ratio *y* = 2.93 are shown in Figures 9 and 10. It is obvious that the heat transfer and friction factor are increased with decreases of cut depth; this is mainly due to the combined effects of common swirling flow by the twisted tape and turbulence generated by the alternative cuts along the edge of the twisted tape. This effect leads to the destruction of the thermal boundary layer and creating better flow mixing between the fluids at the core and heating wall surface.

**Figure 11:**
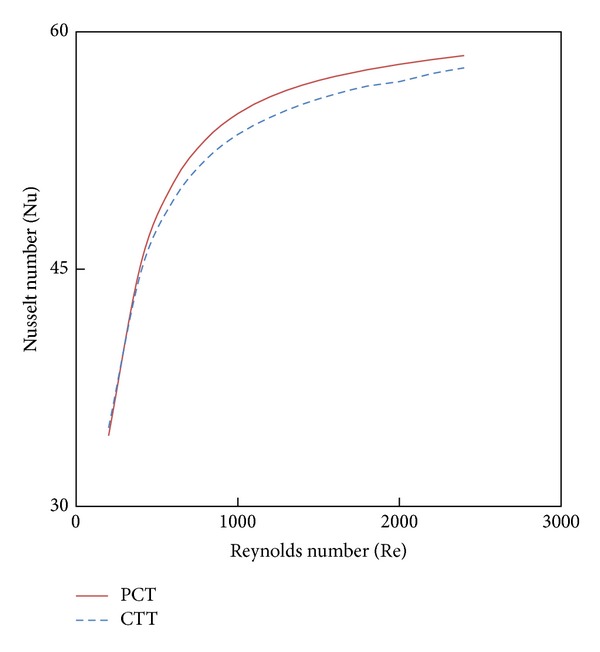
The effect of twist configuration with 4% CuO nanofluid on Nusselt number.

**Figure 12:**
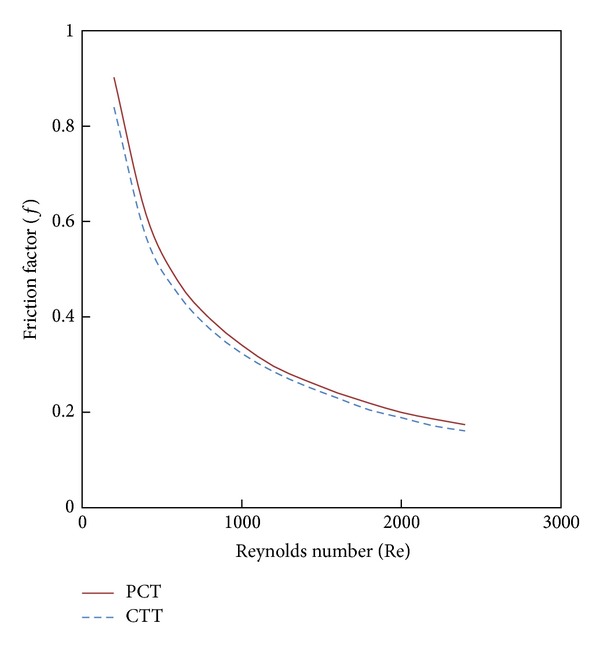
The effect of twist configuration with 4% CuO nanofluid on friction factor.

Figures 11 and 12 show the effect of the new configuration on heat transfer enhancement and friction factor characteristics. Its show that The parabolic-cut twist tape offers heat transfer enhancement better than the classical one with a penalty on the wall shear stress.

## 5. Conclusion

CFD simulation for the heat transfer augmentation in a circular tube equipped with classical and Parabolic-cut twisted tape (PCT) for 2% and 4% volume fraction of CuO nanofluid was carried out using FLUENT version 6.3.26. The data obtained by simulation are matching with the literature correlations of plain tube for validation with the discrepancy of less than ±8% for the Nusselt number and ±10% for friction factor. The results show that the Nusselt number increased with the increase of the nanoparticle volume fraction, Reynolds number, and twist tape decreases. The results also revealed that the twist tape with twist ratio *y* = 2.93 and cut depth (*w* = 0.5 cm) was more dominant than those of (*w* = 1 and 1.5 cm) for all the Reynolds number. Furthermore, the Parabolic-cut twisted tape (PCT) with 4% CuO nanofluid offers about 10% more enhancement of the Nusselt number with significant increases in friction factor than that of Classical twisted tape.

## Figures and Tables

**Table 1:** Thermophysical properties of water and CuO nanofluids.

Fluid	Nanofluids properties			
	Density (kg/m^3^)	Specific heat (J/kg K)	Thermal conductivity (W/m K)	Viscosity (Pa s)
Water	997	4180	0.6096	0.000693
CuO	6500	533	17.6	—
Water + 2% CuO	1017.1	3751.9	0.6797	0.00116
Water + 4% CuO	1217.1	3401.1	0.7163	0.00147

**Table 2:** Thermophysical properties of the materials.

Materials	Density Kg/m^3^	Specific heat J/kg K	Thermal conductivity W/m K
Steel	8030	502.48	16.27
Aluminium	2719	871	202.4

**Table 3:** Numerical values of the parameters used for simulation.

Mass flow rate (Kg/s)	Heat flux (W/m^2^)
0.00384	782.9275132
0.00769	1565.855026
0.01153	2348.78254
0.01537	3131.710053
0.01922	3914.637566
0.02306	4697.565079
0.02690	5480.492592
0.03074	6263.420105
0.03459	7046.347619
0.03843	7829.275132

## Nomenclature

CuO: Copper oxide
*Cp*: Specific heat of the fluid, J/kg K
*dp*: Nanoparticle diameter, nm
*E*: Energy component in energy equation
*F*: Force component in momentum equation, N
*f*: Friction factor
*g*: Acceleration due to gravity, m/s^2^
*k*_eff_: Thermal conductivity in energy equation, W/m K
*m*: Mass flow rate of fluid, kg/s
*Re*: Reynolds number based on internal diameter of the tube, dimensionless
Nu: Nusselt number, dimensionless
*p*: Pressure component in momentum equation, N/m^2^
*S*_*m*_: Accumulation of mass, Kg
*S*_*h*_: Accumulation of energy, J
*T*: Temperature, °C
*v*: Velocity component in momentum equation, m/s
*y*: Twist ratio, dimensionless.

### Greek Symbols

*ρ*: Density component in governing equations
${\vec{\tau }}_{\text{eff}}$: Stress component in momentum equation, N/m^2^.
